# Ferroptosis and Cancer: Complex Relationship and Potential Application of Exosomes

**DOI:** 10.3389/fcell.2021.733751

**Published:** 2021-09-08

**Authors:** Shuang Wu, Tianye Li, Weiwei Liu, Yongye Huang

**Affiliations:** ^1^College of Life and Health Sciences, Northeastern University, Shenyang, China; ^2^Department of Oral and Maxillofacial Surgery, Hospital of Stomatology, Jilin University, Changchun, China; ^3^Jilin Provincial Key Laboratory of Tooth Development and Bone Remodeling, Changchun, China

**Keywords:** ferroptosis, apoptosis, cancer, cell death, exosomes

## Abstract

Cell death induction has become popular as a novel cancer treatment. Ferroptosis, a newly discovered form of cell death, features regulated, iron-dependent accumulation of lipid hydroperoxides. Since this word “ferroptosis” was coined, numerous studies have examined the complex relationship between ferroptosis and cancer. Here, starting from the intrinsic hallmarks of cancer and cell death, we discuss the theoretical basis of cell death induction as a cancer treatment. We review various aspects of the relationship between ferroptosis and cancer, including the genetic basis, epigenetic modification, cancer stem cells, and the tumor microenvironment, to provide information and support for further research on ferroptosis. We also note that exosomes can be applied in ferroptosis-based therapy. These extracellular vesicles can deliver different molecules to modulate cancer cells and cell death pathways. Using exosomes to control ferroptosis occurring in targeted cells is promising for cancer therapy.

## Introduction

### ROS, A Hint for Cancer Therapy

Cancer has become one of the major threats to human health. A report estimated that in 2021 in the United States, there will be 1,898,160 new cancer cases and 608,570 cancer deaths (Siegel et al., 2021). Although the cancer mortality rate has decreased in recent years, access to healthcare has also decreased due to the COVID-19 pandemic, which has led to hampered cancer diagnosis and treatment (Siegel et al., 2021). As widely applied chemoradiotherapy is showing its drawbacks, such as frequent resistance and toxic side effects, cell death induction is becoming increasingly popular for developing novel cancer treatment.

Common forms of cell death, such as apoptosis, autophagy, and necroptosis, are all related to reactive oxygen species (ROS) and are regulated by ROS. For example, ROS can facilitate the extrinsic apoptosis pathway through negative regulation of the cellular FLICE-inhibitory protein (Wang et al., 2008) and can induce intrinsic apoptosis pathways by triggering quick release of Cyt-c (Madesh and Hajnoczky, 2001) and regulating the Bcl-2 protein family (Burlacu, 2003). Evidence shows that ROS generated from ETC and NOX can regulate several pathways that mediate autophagy induction (Li et al., 2011), and AMPK can be activated by AMPK kinase after H_2_O_2_ treatment, which also results in autophagy induction (Choi et al., 2001). In addition, ETC- and NOX-derived ROS are involved in necroptosis facilitation (Dixon and Stockwell, 2014). Evidence of cell death regulation, mediated by ROS, can also be found in ferroptosis and chemosensitization (Galadari et al., 2017).

Given the strong relationship between ROS and cell death, regulating ROS generation upward or controlling oxidative defense downward has become central for new cancer treatments, which have been enhanced by the finding that cancer cells process a higher level of ROS than do healthy cells (Galadari et al., 2017). A higher level of ROS, partly attributed to defective mitochondrial oxidative metabolism (Tafani et al., 2016), can lead to two opposite outcomes: the promotion of cancer and its suppression. Cancer suppression occurs because an elevated ROS level promotes various cell death processes, as mentioned above. Cancer promotion occurs because an elevated ROS level does the following:

**(a)** facilitates tumorigenesis through damaging or modifying cellular proteins, DNA, and lipids, leading to activation or inhibition of various tumorigenesis related signaling cascades (Tafani et al., 2016);

**(b)** promotes angiogenesis by mediating the proliferation, migration, and tube formation of endothelial cells (Potente et al., 2011) or by modulating various vascular endothelial growth factors;

**(c)** contributes to invasion and metastasis through active involvement in essential events including modulating signaling kinases and the cytoskeleton (Tochhawng et al., 2013); and

**(d)** participates in chemoresistance (Ledoux et al., 2003).

Cancer cells exhibiting a greater ROS level display increased activity of antioxidant enzymes, which help create a homeostasis for cell surviving. Therefore, it would be valuable to develop therapeutic strategies to break the redox homeostasis in cancer cells and activate cell death pathways to limit cancer progression. There are two possible approaches: the first is to decrease intracellular ROS. This can be done by, for example, hindering mitochondrial ETC and the activation of NOX, thus inhibiting ROS generation. This technique has been demonstrated in several cancer cell lines and has been proven to be beneficial. A study induced apoptosis in PANC-1 pancreatic cancer cells using diphenylene iodonium, which suppressed ROS generation through inhibiting NOX4 (Mochizuki et al., 2006). The opposite strategy consists of increasing the ROS to a toxic level and thus triggering cell death pathways. Researchers report that piperlongumine, a natural small molecule, can selectively induce ROS-dependent cell death in cancer (Chen et al., 2014). Moreover, glucose metabolism is thought to be related to ROS elimination, and a study has shown that glucose deprivation can induce cytotoxicity in MCF-7/ADR human multidrug-resistant breast cancer cells (Gupta et al., 1997; Lee et al., 1997).

### In This Review

**FIGURE 1:**
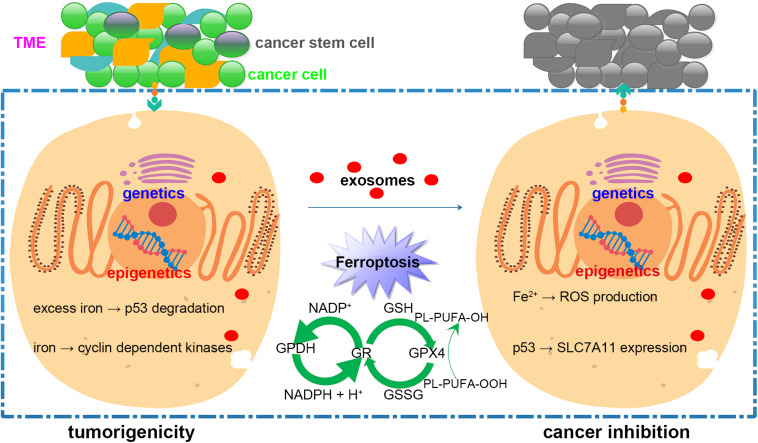
The schematic diagram of modulating ferroptosis in cancer inhibition based on an exosome delivery system. Cancer development and treatment efficacy are impacted by genetic factors, epigenetic modifications, cancer stem cells, and the tumor microenvironment (TME). Ferroptosis is a double-edged sword in cancer survival because it is regulated by different signaling pathways. Cancer treatment could benefit from using an exosome delivery system to control the onset of ferroptosis.

Cancer therapy based on cell death induction has become an important research topic, and ferroptosis, a newly discovered form of cell death, has gained general attention. In this paper, we review the literature on ferroptosis and its relationship with cancer from different perspectives, including proto-oncogene and tumor suppressor gene, epigenetics, cancer stem cells (CSCs), and the tumor microenvironment (TME). Based on the new insights into cancer treatments using cell death induction, we believe ferroptosis to be a promising candidate for cancer treatment. As numerous molecules, ranging from RNAs to plant-derived natural compounds, have been demonstrated to have a therapeutic effect on cancer *via* the induction of ferroptosis-like cell death, drug delivery, which is a critical step in the application of ferroptosis as a cancer treatment, is still being discussed. Given various advantages, such as easy tissue penetration, low toxicity, and low immunogenicity, exosomes are believed to be a reliable drug delivery system able to selectively target specific cells (Figure 1). Here, we provide a simple overview of exosomes and their potential applications in ferroptosis-based therapy.

## Ferroptosis and Cancer

A newly discovered form of cell death, different from apoptosis, autophagy, and necroptosis, called ferroptosis has recently gained recognition for use in cancer treatment. Ferroptosis, a word coined in 2012 (Dixon et al., 2012), is a form of regulated cell death characterized by iron-dependent accumulation of lipid hydroperoxides to lethal levels. It was first used to describe a cell death process induced by a small molecule called erastin, which inhibits the intake of cystine, resulting in glutathione depletion and inactivation of the phospholipid potentially toxic lipid peroxidase 4 (GPX4) (Yang et al., 2014). GPX4 converts lipid hydroperoxide, which is potentially toxic, to a non-toxic form of lipid alcohol (Ursini et al., 1982). Therefore, inactivation or inhibition of the enzyme GPX4 triggers overwhelming lipid peroxidation that causes iron-dependent cell death. Regulation of ferroptosis can be achieved generally by interfering with iron metabolism and ROS metabolism, and the ferroptosis process can be suppressed by iron chelators, lipophilic antioxidants, lipid peroxidation, and the depletion of polyunsaturated fatty acids and correlates with the accumulation of lipid-peroxidation markers (Stockwell et al., 2017).

For the successful application of ferroptosis in cancer treatment, a more concrete understanding of ferroptosis and cancer is needed. The following section contains a review of recent research on ways in which ferroptosis interacts with cancer, especially as regards cancer-related genes, epigenetics, the TME, and so on, and provides a short review of research on ferroptosis regulation and its application to cancer treatment.

## Ferroptosis and Cancer Genes

### RAS

The RAS family of small GTPase, including HRAS, NRAS, and KRAS, is closely related to ferroptosis since the two most well-known ferroptosis inducers, erastin (eradicator of RAS and ST) and RSL3 (RAS-selective Lethal 3), are technically oncogenic RAS-selective lethal small molecules (Yagoda et al., 2007; Yang and Stockwell, 2008). The relationship between ferroptosis and RAS has been carefully investigated by numerous studies (Table 1). For example, researchers have found that HRAS^*V*12^ expressing cancer cells are electively sensitive to ferroptosis, and KRAS silencing in KRAS mutant Calu-1 cells strongly reduces erastin sensibility (Yagoda et al., 2007). A possible explanation might be that constitutive RAS pathway activity promotes TFRC (a gene related to iron metabolism) expression while suppressing the expression of iron storage proteins. However, more evidence for the relation between RAS mutation and erastin sensibility cannot be found in some cancer cell lines (Yang et al., 2014). In contrast, RMS13 rhabdomyosarcoma cells that overexpress HRAS, KRAS, or NRAS are resistant to erastin and RAL3 (Schott et al., 2015), which means RAS does affect erastin sensitivity, while the oncogenic RAS pathway is not the sole determinant of ferroptosis sensitivity (Dixon and Stockwell, 2019). Other research has shown that, in an NRAS^*Q*61*L*^ expressing HL-60 cell line, high mobility group box 1 (HMGB1) is an essential regulator of erastin-induced ferroptosis (Ye et al., 2019). ADP Ribosylation Factor 6 (ARF6), a part of the RAS superfamily, facilitates high sensitivity to RSL3-induced lipid peroxidation (Ye et al., 2020). Reports have shown that oncogenic RAS induces rapid increase of ROS partly through upregulating NOX1 (Irani et al., 1997; Mitsushita et al., 2004). In mice with KRAS-driven pancreatic ductal adenocarcinoma (PDAC), high iron diets and GPX4 depletion, which results in 8-OHG release, lead to macrophage infiltration and activation (Dai E. et al., 2020).

**TABLE 1:** Summary of ferroptosis associated oncogenes and tumor suppressors.

**Gene**	**Target**	**Regulatory direction for target**	**Effect to ferroptosis (references)**
HRAS^*V*12^	iron metabolism related genes	activation	reduce erastin sensibility (Yagoda et al., 2007; Yang et al., 2014)
HRAS^*V*12^	iron storage proteins	inhibition	enhance ferroptosis (Yagoda et al., 2007; Yang et al., 2014)
HRAS^*V*12^	GSH system	activation	reduce erastin sensibility (Schott et al., 2015)
NRAS^*V*12^	GSH system	activation	reduce erastin sensibility (Schott et al., 2015)
KRAS^*V*12^	GSH system	activation	reduce erastin sensibility (Schott et al., 2015)
ARF6	ACSL4	inhibition	enhance RSL3 sensibility (Ye et al., 2020)
p53^*R*237*H*^	NRF2/SLC7A11	regulation	suppress cystine/glutamate antiporter (Sasaki et al., 2002; Habib et al., 2015; Liu D.S. et al., 2017)
p53^*R*175*H*^	NRF2/SLC7A11	regulation	suppress cystine/glutamate antiporter (Sasaki et al., 2002; Habib et al., 2015; Liu D.S. et al., 2017)
p53^3*KR*^	SLC7A11	inhibition	suppress cystine/glutamate antiporter (Jiang et al., 2015)
p53	SLC7A11	inhibition	suppress cystine/glutamate antiporter (Sasaki et al., 2002; Habib et al., 2015; Liu D.S. et al., 2017)
p53	p53-SAT1-ALOX15 axis	activation	enhance ferroptosis (Chu et al., 2019)
p53	GLS2	activation	enhance GSH generation (Hu et al., 2010)
p53	DPP4	regulate the localization and activity	inhibit ferroptosis (Xie et al., 2017)
p53	CDKN1A	activation	delay the onset of ferroptosis (Tarangelo et al., 2018)
MDM2 and MDMX	p53	inhibition	enhance ferroptosis (Venkatesh et al., 2020)
Myc	EGLN1-HIF-1α-LSH-WDR76 axis	activation	inhibit ferroptosis (Jiang et al., 2017)
Myb	CDO1-GPX4 axis	suppress CDO1, and promote GPX4	inhibit ferroptosis (Prouse and Campbell, 2012; Hao et al., 2017)
Src	GPXs	Src acts as the target for GPXs	participate in ferroptosis regulation (Wei J. et al., 2020)
Src	ACSL4	inhibition	inhibit ferroptosis (Brown et al., 2017)

### p53

Because of its role in cell cycle arrest, senescence, and apoptosis, as well as for its interesting role in metabolism, oxidative responses, and ferroptosis, p53 has long been an important focus of research. p53’s essential function of survival promotion is confirmed by the fact that cells are more sensitive to ferroptosis after p53 depletion through CRISPR/Cas9 (Tarangelo et al., 2018). By antagonizing p53 activity, such as the O-GlcNAcylated c-Jun (the first discovered oncogenic transcription factor), cell death can be prevented (Eferl et al., 2003). Recently, p53-related signal pathways have been shown to modulate ferroptosis in the following ways.

Research has shown that p53 can suppress the expression of SLC7A11, a key component of the cystine/glutamate antiporter (X_*c*_^–^ system), leading to the inhibition of cystine uptake and sensitization to ferroptosis. For example, the p53 mutants p53^*R*237*H*^ and p53^*R*175*H*^ promote sensitivity to ferroptosis-like cell death, most likely through the combination of p53 mutants with NRF2 and through the suppression of the NRF2-dependent transactivation of SLC7A11 together with other antioxidant genes that oppose ferroptosis (Sasaki et al., 2002; Habib et al., 2015; Liu D.S. et al., 2017). Moreover, overexpression of SLA7A11 in human tumor suppresses ROS-induced ferroptosis and inhibits p53^3*KR*^-mediated tumor growth suppression in xenograft models. Though mutant p53^3*KR*^ effectively downregulates SLC7A11, it does not affect other p53 target genes involved in cell cycle regulation or apoptosis (Jiang et al., 2015). In contrast, mutant p53^4*KR*98^ is unable to reduce SLC7A11 expression (Wang et al., 2016).

As a transcription target of p53, the activity of spermidine/spermine N^1^-acetyltransferase 1 (SAT1), the rate-limiting enzyme in polyamine catabolism, induces lipid peroxidation and sensitizes cells to undergo ferroptosis and the deletion of SAT1 suppresses p53 and p53^3*KR*^-mediated ferroptosis. However, while p53 modulates SLC7A11 expression, the expression and activity of SLC7A11 and GPX4 are not associated with SAT1, and only ferrostatin-1 can inhibit ROS-induced ferroptosis in Tet-on cells (Ou et al., 2016). Research has also found that SAT1 induction is associated with the expression of arachidonate 15-lipoxygenase (ALOX15), which is essential in p53-mediated ferroptosis (Chu et al., 2019). Unfortunately, the p53–SAT1–ALOX15 axis has not been fully explained.

The metabolism of glutamine, one of the essential components of ferroptosis, is catalyzed by cytosolic glutamine aminotransferases or by mitochondrial glutaminases (Gao et al., 2015; Altman et al., 2016). The expression of glutaminase 2 (GLS2), which has been identified as a transcriptional target of p53, mediates oxygen consumption, mitochondrial respiration, and ATP generation in cancer cells. Based on the evidence that GLS2 facilitates GSH production in several cancer cell lines, GLS2 is recognized as a negative regulator of ferroptosis (Hu et al., 2010).

Colorectal cancer (CRC) caused by a number of genetic disorders, including KRAS mutation, p53 mutation, and p53 depletion, which provides additional evidence for the survival-promoting function of p53. Interestingly, this p53 function might partly be achieved by modulating ferroptosis. Research has found that p53 can inhibit ferroptosis by modulating the localization and activity, but not expression, of dipeptidyl peptidase-4 (DPP4), leading to survival promoting functions. This process occurs through a post-translational interaction with protease DPP4, which strengthens membrane lipid peroxidation in a protease-independent way *via* interaction with an ROS-generating NOX (Xie et al., 2017).

Cyclin dependent kinase inhibitor 1A (CDKN1A/p21), also known as p21^*WAF*1/Cip1^, is a key mediator of p53-dependent cell cycle arrest after DNA damage (Abbas and Dutta, 2009). A recent study has shown that the expression of CDKN1A, mediated by p53, delays the onset of ferroptosis in response to subsequent cystine deprivation in cancer cells (Tarangelo et al., 2018). As two negative regulators of p53, MDM2 and MDMX facilitate ferroptosis with or without p53, most likely by altering the lipid profile of cells (Venkatesh et al., 2020), which is confirmed by evidence that with the treatment of MDM2 inhibitor nutlin-3, p53 expression increases and leads to the suppression of X_*c*_^–^ system inhibitor-induced ferroptosis in HT-1080 cells (Tarangelo et al., 2018). The function of CDKN1A in cell cycle arrest, which is unable to trigger ferroptosis, is primarily achieved by binding to and suppressing the kinase activity of the cyclin-dependent kinases (CDKs) (Abbas and Dutta, 2009).

Interestingly, a recent study reported that retention of p53 in the nucleus, mediated by the interaction of long non-coding RNA (lncRNA) P53RRA, and Ras GTPase-activating protein-binding protein 1 (G3BP1), leads to cell cycle arrest, apoptosis, and ferroptosis. This is because p53 is displaced from the G3BP1 complex (Mao et al., 2018).

### Myc

Studies tend to view Myc proteins as transcriptional factors that exert tumorigenesis functions by activating and suppressing target genes (Lutz et al., 2002). Evidence has shown a relationship between Myc and ferroptosis. A recent study reported that egl nine homolog 1 (EGLN1) and Myc activate lymphoid-specific helicase (LSH) expression through HIF-1α, and that LSH suppresses ferroptosis through the interaction with WDR76, leading to the activation of lipid metabolism-associated genes (Jiang et al., 2017). Another study reported that the depletion of VHL, a major tumor suppressor of clear cell renal cell carcinoma (ccRCC), leads to the stabilization of the hypoxia inducible factors HIF-1α and HIF-2α. This paper also found that exogenous expression of pVHL can revert ccRCC cells to an oxidative metabolism and a state of insensitivity to ferroptosis induction. Myc-dependent tumor growth in mouse models can be inhibited by GSH synthesis suppression (Miess et al., 2018). A newly identified oncogene, DJ-1, displays ferroptosis resistance and can synergistically transform mouse NIH3T3 cells together with activated GTPase HRAS and MYC proto-oncogene (c-Myc) (Jiang et al., 2020).

### Myb

Members of the Myb family are found in all eukaryotic lineages, the function of which is to regulate fundamental cellular processes, metabolism, and cellular differentiation (Prouse and Campbell, 2012). Evidence shows that c-Myb is involved in ferroptosis through a cysteine dioxygenase 1 (CDO1)–GPX4 axis. Silencing CDO1 leads to suppression of erastin-induced ferroptosis *in vitro* and *in vivo*, and inhibition of CDO1 restores cellular GSA levels, which prevents ROS generation. This paper demonstrates that c-Myb transcriptionally regulates CDO1 and inhibition of CDO1 expression upregulates GPX4 (Hao et al., 2017).

### SRC

Cellular SRC (c-SRC), the product of the SRC gene, is involved in tumorigenesis, invasion, and the metastatic phenotype (Alper and Bowden, 2005). A recent study has found that the SRC gene is one of the targeting sites of GPX4, the differential expression of which regulates cell proliferation, cancer progression, apoptosis, and ferroptosis (Wei J. et al., 2020). Another report demonstrated that, mediated by α6β4 integrin, the activation of SRC and STAT3 could inhibit ACSL4 expression, leading to the protection of adherent epithelial and carcinoma cells form erastin-induced ferroptosis (Brown et al., 2017). This is partly because ferroptosis cannot be triggered while there is a lack membranes enriched by ACSL4-mediated long polyunsaturated fatty acids. It was also proved that matrix-detached epithelial and cancer cells cluster spontaneously through a pathway involved with Nectin-4 (also known as cell adhesion protein PVRL4), the process of which sustains GPX4 expression and buffers against lipid peroxidation by stimulating the PVRL4/α6β4/Src axis signal pathway (Brown et al., 2018).

### Rb

The retinoblastoma (Rb) protein is the founding member of a protein family that exerts a strong regulatory function on the transcription of various genes in eukaryotes (Knudsen and Knudsen, 2008). Ferroptosis in hepatocellular carcinoma can be promoted, resulting in two or three times more cell death, by sorafenib treatment combining with Rb knockdown using RNA interference (Louandre et al., 2015).

## Ferroptosis and Epigenetics

### Non-coding RNA

Non-coding RNAs (ncRNAs) are RNAs in the transcriptome and will not be translated into proteins. They are identified as several subfamilies based on their molecular size and shape, including long non-coding RNAs (lncRNAs), microRNAs (miRNAs), small nuclear RNAs (snRNAs), and small interfering RNAs (siRNAs) (Hombach and Kretz, 2016). Non-coding RNAs are increasingly regarded as essential regulators of ferroptosis in cancer and a better understanding of them can provide novel ideas for cancer treatment.

MiRNAs exhibit functions by binding to the 3′-untranslated regions of their target mRNAs and thus prevent the expression process (Majidinia et al., 2020). Studies have demonstrated that miRNAs regulate ferroptosis through direct and indirect approaches. For example, miR-7-5p inhibits ferroptosis by downregulating mitoferrin and reducing iron levels in radio-resistant cells (Tomita et al., 2019). miR-6852, which is regulated by lncRNA linc00336, can inhibit lung cancer progression by promoting ferroptosis. Besides direct regulation, evidence shows that miRNAs affect the metabolism of GSH, a scavenger of ROS that protects lipid membrane (Hsu et al., 2019). For instance, miR-18a and miR-218 downregulate GSH levels in hepatocellular carcinoma and bladder cancer separately by targeting GCL (Anderton et al., 2017; Li P. et al., 2017), while miR-152 and miR-155 decrease GSH levels in hepatocellular carcinoma and lung cancer separately by targeting GST (Huang et al., 2010; Lv et al., 2016), the general pathway by which miRNAs modulate GSH level. GST can be targeted and modulated by various miRNAs, including miR-92b-3p, miR-124, miR129-5P, miR-130b, miR-133a/b, miR-144, miR-153-1/2, miR-186, miR-302c-5p, miR-513a-3p, miR-590-3p/5p, miR-36645p, miR-3714, and let-7a-5p (Zhang et al., 2020e). In the meantime, iron metabolism mainly includes the interaction between transferrin (TF) and TF receptor (TFR), which can also be regulated by miRNAs. For example, in CRC and hepatocellular cancer, TFR can be targeted by miRNAs including miR-22, miR-31, miR-141, miR-145, miR-152, miR-182, miR-200a, miR-320, miR-758, and miR19463–65, resulting in a disruption between TF and TFR and the following iron importing process (Zhang et al., 2020e). Moreover, iron can regulate miRNA levels. Levels of miR-107 and miR-125b can be suppressed by iron in hepatocellular carcinoma (Lobello et al., 2016; Zou et al., 2016), while levels of miR-146a and miR-150 can be increased by iron (Sriramoju et al., 2015; Lobello et al., 2016), which might be due to iron’s induction of excess ROS (Zhang et al., 2020e). Moreover, miRNAs regulate the NRF2 pathway through by targeting Kelch-like ECh-Associated Protein 1 (KEAP1) and NRF2 mRNAs (Zhang et al., 2020e).

LncRNAs generally serve as regulators of transcription factors in the nucleus or as sponges of miRNAs in the cytoplasm (Wu et al., 2020). The silence of lncRNA ZFAS1, which acts as a ceRNA and sponge for miR-150-5p, suppresses ferroptosis by downregulating SLC38A1 (Yang Y.N. et al., 2020). Besides the relationship between linc00336/miR-6852 and lncRNA P53rra/G3BP1 mentioned above, lncRNAs modulate ferroptosis indirectly by targeting ferroptosis-associated factors (Table 2). A study reported that the reduction of lncRNA ROR leads to reduced GST expression in breast cancer (Li Y.H. et al., 2017), and silencing lncRNA Neat1 contributes to an increase of GST (Wang et al., 2018). Other studies have shown that lncRNAs are associated with iron metabolism and that silencing lncRNA PVT1 suppresses TFR expression and obstructs iron intake *via* miR-150 (Xu et al., 2018). Evidence also shows that lncRNAs affect the expression of NRF2 by directly and indirectly modulating KEAP1 levels, while NRF2 is associated with lncRNA regulation (Zhang et al., 2020e). Besides the above factors, ROS levels can be regulated by lncRNAs. For instance, decreased expression of lncRNA H19 increases ROS *via* the MAPK/ERK signaling pathway (Ding et al., 2018), while the reduction of lncRNA growth arrest specific 5 in melanoma enhances intracellular ROS (Chen et al., 2019). Increased levels of lncRNA GABPB1-AS1 downregulate the peroxiredoxin-5 peroxidase gene and ultimately inhibits the antioxidant capacity of cells (Qi et al., 2019).

**TABLE 2:** lncRNAs participate in the regulation of ferroptosis.

**lncRNA**	**Target**	**Regulatory direction for target**	**Effect to ferroptosis (references)**
ZFAS1	SLC38A1	activation	enhance ferroptosis (Yang Y.N. et al., 2020)
PVT1	TFR	inhibition	block iron intake (Xu et al., 2018)
H19	MAPK/ERK signaling	regulation	modulate ROS production (Ding et al., 2018)
GABPB1AS1	peroxiredoxin-5 peroxidase	inhibition	decrease antioxidant capacity (Qi et al., 2019)
OIP5-AS1	miR-128-3p/SLC7A11 signaling	sponge	inhibit ferroptosis (Zhang Y. et al., 2021)
NEAT1	ACSL4	regulation	regulate ferroptosis and ferroptosis sensitivity (Wu and Liu, 2021)
LINC00618	lymphoid-specific helicase (LSH)	attenuate LSH to recruit to the promoter regions of SLC7A11	increase ROS and iron, accelerate ferroptosis (Wang Z. et al., 2021)
MT1DP	miR-365a-3p/NRF2 axis	stabilize miR-365a-3p to modulate NRF2 expression	increase intracellular ferrous iron (Gai et al., 2020)
LINC00336	ELAVL1	binding	inhibit ferroptosis (Wang M. et al., 2019)
P53RRA	G3BP1	binding	cytosolic P53RRA-G3BP1 interaction displaces p53 from a G3BP1 complex, induce ferroptosis (Mao et al., 2018)

Other ncRNAs, such as circRNAs RNAs, rRNAs, piRNAs, snRNAs, and snoRNAs, also interact with ferroptosis in various cancer types (Table 3). For circRNAs, circIL4R facilitates tumorigenesis and prevents ferroptosis by regulating the miR-541-3p/GPX4 axis (Xu et al., 2020a). The reduction of circ-TTBK2 delays proliferation and invasion of glioma cells by regulating the miR-761/ITGB8 axis and triggering ferroptosis (Zhang et al., 2020b). Another study reports that circRNA clARs regulate ferroptosis through interacting with the RNA binding protein ALKBH5 (Liu et al., 2020). A recent study has shown that reduction of circ0008035 enhances the anticancer effects of erastin and RSL3 by increasing iron accumulation and lipid peroxidation (Li C. et al., 2020). Moreover, studies revealed that tRNA upregulates ferroptosis by suppressing GSH biosynthesis in a GPX4-independent pattern. However, in contrast, tRNAs can also downregulate ferroptosis by enhancing the antioxidant defense system (Zhang et al., 2020e). Moreover, rRNAs, piRNAs, snRNAs, and snoRNAs were recently found to be involved in ferroptosis-associated pathways (Zhang et al., 2020e).

**TABLE 3:** circRNAs modulate the induction of ferroptosis in cancer.

**circRNA**	**Target**	**Regulatory direction for target**	**Effect to ferroptosis (references)**
IL4R	miR-541-3p/GPX4 axis	sponge	inhibit ferroptosis (Xu et al., 2020a)
TTBK2	miR-761/ITGB8 axis	sponge	inhibit ferroptosis (Zhang et al., 2020b)
clARs	ALKBH5	interaction	regulate ferroptosis (Liu et al., 2020)
KIF4A	circKIF4A-miR-1231-GPX4 axis	sponge	inhibit ferroptosis (Chen W. et al., 2021)
circ0097009	circ0097009/miR-1261/SLC7A11 axis	sponge	regulate ferroptosis (Lyu et al., 2021)
RHOT1	miR-106a-5p/STAT3 axis	sponge	inhibit ferroptosis (Zhang H. et al., 2020a)
EPSTI1	miR-375/409-3P/515-5p-SLC7A11 axis	sponge	regulate ferroptosis (Wu et al., 2021)
ABCB10	miR-326/CCL5 axis	sponge	regulate ferroptosis (Xian et al., 2020)
TTBK2	miR-761/ITGB8 axis	sponge	regulate ferroptosis (Zhang et al., 2020b)

### Methylation

Various studies have revealed the function of DNA or protein methylation in tumor progression, ROS metabolism, and iron metabolism; however, despite being one of the most common molecular modification in epigenetics, the direct relationship between methylation and ferroptosis has not been fully discussed.

Some studies demonstrated the indirect regulation of ferroptosis *via* DNA and protein methylation. For example, lymphoid-specific helicase (LSH), a DNA methylation modifier, can activate lipid metabolism-associated genes to inhibit ferroptosis by interacting with WDR76 (Jiang et al., 2017), and together with another W40 protein DCAF8, they function as a crucial nexus in epigenetic regulation of ferroptosis, controlling LSH degradation by adapted oxidative damage sensing through DNA hydroxymethylation (Huang et al., 2020). The silencing of the DNA methylation of the elongation of very long-chain fatty acid protein 5 (ELOVL5) and fatty acid desaturase 1 (FADS1) leads to ferroptosis resistance, and these two enzymes are usually upregulated in mesenchymal-type gastric cancer cells (Lee J.-Y. et al., 2020). Besides, GPX4 methylation is also reported to be related to ferroptosis regulation. For example, homocysteine (Hcy), an amino acid involved in DNA methylation, facilitates GPX4 methylation that leads to upregulation of oxidative stress and ferroptosis in nucleus pulposus (Zhang et al., 2020c). Another study reported that the increased expression of GPX4 in cancer tissues might be partly attributed to a lower level of DNA methylation and histone acetylation (Zhang et al., 2020d). A report has shown that KDM3B, a histone H3 lysine 9 demethylase, can protect against erastin-induced ferroptosis and is thus considered a potential epigenetic regulator of ferroptosis (Wang et al., 2020). Meanwhile, the expression of iron metabolism-associated genes, including TRFC, FTH1, and FTL, can be modulated by the epigenetic silencing of the iron-responsive element binding protein 2 (IREB2) (Dixon et al., 2012), while other perturbations of mechanisms, including acetylation and methylation, have been observed to regulate iron metabolism in cancer cells by controlling transcript encoding proteins (Manz et al., 2016).

Some ferroptosis regulation pathways have been found recently in which tumor-associated factors are usually involved. For instance, in head and neck cancer cells, diminution of the hypermethylation of CDH1 results in increased E-cadherin expression and decreased ferroptosis susceptibility (Lee J. et al., 2020); this work also provides evidence that epithelial–mesenchymal transition (EMT) promotes ferroptosis *via* epigenetic regulation pathways. The lower promoter methylation of GPX1, a member of the GPX family that interact with oxidative stress, results in high expression levels of GPX1 in some cancer cell lines (Wei R. et al., 2020). Another study shows that JQ1 can inhibit BRD4 expression and ultimately induce ferroptosis through two pathways, either by inhibiting the histone methylase G9a or by activating the histone deacetylase SIRT1, which can recognize the acetylation site and recruit transcriptional factors (Sui et al., 2019).

### Acetylation

A widely occurring post-translational modification, acetylation plays a role in ferroptosis mainly through direct and indirect interaction with ferroptosis regulators. The acetylation of genes and proteins involved in ferroptosis is reported to regulate iron-dependent cell death. For example, an acetylation defect is observed in mutant p53^3*KR*^, which indirectly inhibits cysteine absorption and reduces GSH consumption, leading to lipid peroxidation and ferroptosis (Jiang et al., 2015). Acetylation absence in the mouse p53 K98 site and on other positions in the DNA-binding domain can result in the loss of tumor suppression functions in xenografts and ferroptosis (Wang et al., 2016). Another study reported that RSL3 promotes the protein expression and acetylation of ALOX12, the key protein in initiating membrane phospholipid oxidation (Wang Y. et al., 2021). Indirect regulation is also observable. It has been reported that suppression of EMT mediated by histone deacetylase SIRT1 gene silencing or pharmacological inhibition consequently decreases ferroptosis, which further suggests that EMT promotes ferroptosis through epigenetic regulation pathways (Lee J. et al., 2020). Moreover, acetylation of HMGB1, a damage-associated molecular pattern molecule (DAMP), is released by ferroptosis cells in an autophagy-dependent manner (Wen et al., 2019).

### Ubiquitination

Ubiquitination is a post-translational modification involved in essential host processes that has been reported to regulate ferroptosis epigenetically. The most common regulation pathway involves interaction with SLC7A11, which is essential in the X_*c*_^–^ system. Evidence suggests that the deubiquitinase OTUB1, usually overexpressed in cancers, replicates the ferroptosis process and promotes tumor development by stabilizing the cystine transporter SLC7A11 (Gan, 2019). Once deubiquitinase is suppressed, caspase-dependent apoptosis and GPX4-degradation-dependent ferroptosis is activated, contributing to the accumulation of ubiquitination proteins that facilitates cell death (Yang L. et al., 2020). The tumor suppressor BAP1, an H2A deubiquitinating enzyme, can reduce SLC7A11 expression by inhibiting H2A ubiquitination (H2Aub) on the SLC7A11 promoter, thus controlling ferroptosis (Zhang Y.L. et al., 2019). Another study shows that p53 may also be involved in ubiquitination-dependent regulation of ferroptosis. For example, p53 decreases H2B ubiquitination occupancy in the SLC7A11 gene regulatory domain and represses its expression (Wang Y. et al., 2019). Ubiquitination also regulates ferroptosis by modulating ferritin degradation. In iron deficiency, nuclear receptor coactivator 4 (NCOA4) specifically binds iron-rich ferritin to autophagosomes through FTH1 and transports it to the lysosome for iron release, while NCOA4 can be degraded through ubiquitination, which affects that stability of ferritin. Therefore, suppressing NCOA4 can inhibit the degradation of ferritin and the occurrence of ferroptosis (Capelletti et al., 2020).

## Ferroptosis and Cancer Stem Cells

### Hallmarks of CSCs

CSCs are a small section of tumor cells that possesses the ability to self-renew, initiate tumors, and cause resistance to conventional anticancer agents. Different from regular cancer cells, CSCs have a lower level of ROS, which might contribute to a slower growth rate, reduced oxidative metabolism, and elevated expression of the ROS scavenging system (Bystrom et al., 2014; Ding et al., 2015; Hyewon and Navdeep, 2018). Lipid intake pathways are upregulated in CSCs, providing energy essential for survival, which explains why interference with GPX4 pathways seems to render CSCs sensitive to ferroptosis (Recalcati et al., 2019; Visweswaran et al., 2020). Higher iron levels are another characteristic of CSCs, such that ferroptosis may be a good method for eliminating CSCs, which are less susceptible to classical anticancer apoptosis-inducing agents. Indications of higher iron levels consist of the expression levels of TFR1 and its ligand iron-loaded TF is upregulated in glioblastoma CSCs compared to non-CSCs (David et al., 2015). Furthermore, cellular iron, TFR1, and TF uptake are more robust in breast CSCs compared to non-CSCs (Mai et al., 2017). TFR1 and ferritin are essential for propagation and formation of tumors *in vivo*. On the other hand, forced reduction of intracellular iron reduces the proliferation and tumorigenicity of ovarian CSCs (Basuli et al., 2017). Evidence points to multiple roles of intracellular iron in CSC proliferation and stemness maintenance (Recalcati et al., 2019). For instance, in breast cancer cells, low iron levels are associated with a lower expression of EMT markers (Guo et al., 2015). Iron also mediates the downregulation of E-cadherin, a hallmark of EMT (Brookes et al., 2008).

### Ferroptosis-Based Treatment of Cancer Stem Cells

Higher iron levels do not necessarily relate to ROS levels and ferroptosis, but it has been proven that CSCs are highly sensitive to ferroptosis due to increased expression levels of TFR1, and thus ferroptosis-based treatment and therapeutic interference of iron homeostasis can have a curing effect on cancer (Mai et al., 2017). Notably, recent studies indicate that triggering ferroptosis may specifically kill CSCs; for example, salinomycin can drive ferroptosis-based cell death in breast CSCs (Zhao et al., 2019), and ironmycin, a derivative of salinomycin, can specifically trigger iron accumulation in lysosomes, activating cell death pathways consistent with ferroptosis (Mai et al., 2017). Some small-molecule ferroptotic agents also have the potential to selectively kill breast CSCs (Taylor et al., 2019). The blocking of the lysosomal iron translocation of CSCs by inhibiting the divalent metal transporter 1 (DMT1) leads to iron accumulation and cell death with features of ferroptosis (Turcu et al., 2020). In colorectal CSCs, knockdown or inhibition of SLC7A11 significantly and specifically kills cancer cells and thus attenuates chemoresistance in CRC (Xu et al., 2020c). Besides, two nitroimidazoles (Koike et al., 2020), itraconazole (Xu et al., 2021), and dichloroacetate (Sun et al., 2021) are also proven to have therapeutic potential through promoting ferroptosis in CSCs.

## Ferroptosis and the Tumor Microenvironment

The TME functions as a cradle for tumorigenesis and cancer progression. Understanding the TME and ferroptosis interaction may provide novel and effective anticancer strategies.

A recent study reports that ferroptosis can promote tumor growth by driving macrophage polarization in the TME (Dai E.Y. et al., 2020). Hypoxia is one of the known characteristics of the TME, which is controlled by the hypoxia-inducible factor (HIF) (Labiano et al., 2015). Researchers have found that hypoxia is an essential positive trigger for ferroptosis, and HIF-2α enhances lipid peroxidation while the depletion of HIF-1α decreases sensitivity to ferroptosis (Zou et al., 2019). Moreover, iron metabolism-associated genes, including FTH, TFR1, and SLC11A2, are regulated by hypoxia-responsive elements (HREs) in the promotor region (Li et al., 2019).

### Antitumor Immunity

Ferroptosis is thought to be linked to antitumor immunity. This was first proved by the study that immunotherapy-activated CD8^+^ T lymphocytes can induce ferroptosis in cancer cells by downregulating SLC7A11 and SLC3A2, encoding subunits of system X_*c*_^–^. Technically, this study has shown that tumor cell coculture with IFN-γ-rich supernatant obtained from activated T cells induces lipid peroxidation and ferroptosis (Wang W.M. et al., 2019). Overexpression of ferroptosis suppressor protein 1 (FSP1) or cytosolic GPX4 stimulates the genesis of ferroptosis-resistant CD8^+^ T cells without compromising their function, while the depletion of ferroptosis sensitivity-promoting enzyme acyl-CoA synthetase long-chain family member 4 (ACSL4) protected CD8^+^ T cells from ferroptosis but impaired antitumor CD8^+^ T cell response (Drijvers et al., 2021).

Other studies have demonstrated that cancer cells that have undergone ferroptosis can release high mobility group Box 1 (HMGB1) in an autophagy-dependent manner (Yu et al., 2015; Wen et al., 2019). When HMGB1 is released into the TME because of cancer cell death, it can stimulate the innate immune system by interacting with several pattern recognition receptors (Sims et al., 2010; Yamazaki et al., 2014). Evidence shows that during ferroptosis, tumor cells supply arachidonic acid for eicosanoid synthesis, which can strengthen antitumor immunity (Angeli et al., 2019). Moreover, ferroptosis induction in tumor cells is thought to be related to the release of prostaglandin E2 (PGE2), which facilities the evasion from immune surveillance (Yang et al., 2014).

### Nanoparticles and Immunotherapy

The synergism between ferroptosis and immunomodulation in cancer has been widely investigated in recent decades. On the one hand, TME immunomodulation can trigger macrophage polarization from alternately activated macrophages M2 to classically activated macrophages M1, offering intertumoral H_2_O_2_ for the Fenton reaction (Zanganeh et al., 2016), which effectively generates ROS and triggers lipid peroxidation (Yang and Stockwell, 2016; Sun et al., 2017). On the other hand, ferroptosis in tumor cells can release tumor antigens and generate an immunogenic TME, thus enhancing the immunomodulation response (Zhang F. et al., 2019). Nanoparticles (NPs), which can passively infiltrate tumor tissues because of the enhanced permeability and retention, act as a drug-loading platform with high loading efficiency, and release specific cargos in tumor issues, are gaining recognition in immunotherapy.

Some metal elements are especially popular for their inherent physicochemical properties, and metal-containing nanomaterials are designed for ferroptosis-driven therapy. They can function in different manners, including facilitating Fenton-like reactions, providing hydrogen peroxide, damaging the reducing system, and disturbing cellular communication (Fei et al., 2020). For example, biomimetic magnetosome, composed of an Fe_3_O_4_ magnetic nanocluster with a TGF-β inhibitor loaded inside and a PD-1 antibody anchored on the membrane surface, was developed to promote ferroptosis/immunomodulation synergism in cancer (Zhang F. et al., 2019). MnO_*x*_ nanospikes, as TME-responsive nano-adjuvants and immunogenic cell death drugs, were also designed for cancer nanovaccine-based immunotherapy (Ding et al., 2020). In another study, in which ultrasmall CaO_2_ and Fe_3_O_4_ were co-loaded on to dendritic mesoporous silica NPs, researchers showed that these particles can achieve tumor specialized localization and induction of Fenton reaction, thus triggering ferroptosis (Li and Rong, 2020). The Fe_3_O_4_-PLGA-Ce_6_ nanosystem, which dissociates in acidic TME, and the Fe^2+^-based metal–organic framework, which delivers Fe^2+^ to cancer cells, can also promote the Fenton reaction and facilitate ferroptosis (Xu et al., 2020b; Chen Q. et al., 2021).

Although nanotechnology is increasingly used in cancer treatment, the application of NP-based therapy faces various issues, such as intrinsic immunogenicity and residual cytotoxicity (Shen et al., 2018). In a new approach that has high biocompatibility, low immunogenicity, preferred tumor homing, and high efficiency in cargo delivery, the 30- to 120-nm endocytic lipid bilayer membrane-derived vesicles is attracting attention as a novel drug carrier for ferroptosis induction (Qin and Xu, 2014; Kibria et al., 2018). Attempts have been made to use exosomes as carriers for ferroptosis-inducing drugs to trigger cell death among cancer cells. For example, engineered M1 macrophages, with CCR2 overexpression, are employed as Fe_3_O_4_ NP carriers (Li et al., 2021). Moreover, a well-known ferroptosis inducer, erastin, can be loaded into exosomes labeled with folate and delivered to cancer cells that express the folate receptor to generate ROS and glutathione depletion (Yu et al., 2019).

## Exosomes

Generated from the plasma membrane (Simons and Raposo, 2009), exosomes were first used for carrying clotting suppressors (Wolf, 1967). Since then, these extracellular vesicles have been shown to be secreted by various kinds of cells, including dendritic cells, macrophages, T cells, B cells, mesenchymal stem cells, endothelial cells, epithelial cells, and various cancer cells (Song H. et al., 2021).

### Biogenesis and Composition

Exosomes are generated from late endosomes through several different pathways. Endosomal-sorting complexes required for transport (ESCRTs), which recognize ubiquitylated proteins, are the most characterized one among genesis pathways, while others may involve sphingomyelinases (Trajkovic et al., 2008), sphingosine-1-phosphate, and tetraspanin-enriched domains (Brinton et al., 2015). Four ESCRTs, numbered from 0 to 3, consist of many proteins able to recognize ubiquitinated cargoes. Technically, ESCRT-0 subunits recruit proteins for internalization, such as ubiquitinated proteins and clathrin. ESCRT-1 and ESCRT-2 control the initiation of the budding process and facilitate the enzymatic de-ubiquitination of cargo proteins before the formation of intraluminal vesicles (ILVs). ILVs then gather to form larger membranous vesicles in the intracellular compartment. ESCRT-3 drives membrane invagination and separation (Ha et al., 2016; McGough and Vincent, 2016). According to the genesis process, the composition pattern of exosomes faithfully reflects their parent cells. For proteins displayed on the surface, adhesion molecules, which belong to the tetraspanin and integrin families, are the most abundant. These proteins, which are generally membrane crossing, include CD9, CD63, CD81, and CD82 and regulate processes like fusion, migration, and adhesion. They usually attach to each other or associate with nearby proteins, such as integrins, to form a tetraspanin membrane domain (Farooqi et al., 2018). The major histocompatibility complex II (MHC-II) may be present on the surface of exosomes and is involved in promoting certain T-cell responses (Elena and Myung Soo, 2015). Moreover, tumor-derived exosomes are able to promote cancer cell migration and metastasis, containing various kinds of integrin, such as exosomal integrins α_*v*_β_6_ for prostate cancer (Carmine et al., 2015), α_6_β_4_ and α_6_β_1_ for lung cancer, and α_*v*_β_5_ for liver cancer metastasis (Hoshino et al., 2015). Other protein molecules, such as annexins, flotillin, and GTPases, are associated with lipid fractions on exosomes and serve transportation and fusion functions (Colombo et al., 2014). Besides proteins, lipids are another main component of exosomes, which depend on the type of parent cell plasma membrane. Phosphatidylethanolamines, phosphatidylcholines, phosphatidylinositols, phosphatidylserines, sphingomyelins, lysobisphosphatidic acid (bis-monoacylglycerol phosphate), phosphatidic acid, cholesterol, lysophosphatidylcholines, ceramide, and phosphoglycerides have been found in these membranes (Caroline et al., 2007). The intraluminal composition of the exosomal membrane also depends on the parent cells and particularly on their cytoplasmatic content. Exosomes shuttle through the body, allowing the horizontal transfer of their cargo while fusing with target cells and releasing their content *via* an endocytosis process, and thus participate in various regulation pathways (Escrevente et al., 2011). A wide range of molecules have been found in different cell-derived exosomes, such as heat shock proteins, cytoskeletal proteins, lipids, and enzymes, along with nucleic acid molecules, such as miRNAs, mRNAs, ncRNAs, mitochondrial DNA, and single-strand DNA (Farooqi et al., 2018).

### Exosomes in Cancer and Ferroptosis Regulation

Among the numerous biological roles played by exosomes, their function in cancer is becoming increasingly apparent. A number of studies have revealed that exosomes can regulate the function of target cells by secreting their contents into the TME, using crosstalk, and/or influencing major tumor-related pathways, including EMT, CSCs, angiogenesis, and metastasis involving several cell types (Ha et al., 2016; Wu et al., 2017). Moreover, drug resistance is partly attributed to exosomes, for cancer cells can encapsulate therapeutic drugs in exosomes and transport them out of tumor cells (Arrighetti et al., 2019). Evidence shows that exosomes also overlap with ferroptosis modulation. For example, mesenchymal stromal cells (MSCs) derived from human umbilical cord blood (HUCB-MSCs) tend to significantly inhibit the expression of DMT1 by miR-23a-3p to inhibit ferroptosis (Song Y. et al., 2021). The miR-522 inside exosomes, generated from cancer-associated fibroblasts (CAFs), can block lipid-ROS accumulation by targeting ALOX15 and thus inhibit ferroptosis (Zhang H. et al., 2020a). In a recent study, researchers found that ferroptosis promotes tumor growth by driving macrophage polarization in the TME. One kind of common KRAS mutant, KRAS^*G*12*D*^, is secreted into the TME from tumor cells after succumbing to autophagy-dependent ferroptosis. This extracellular protein is then packaged into tumor-derived exosomes and is absorbed by macrophages, leading to the switch from the M1 phenotype to the M2 phenotype and accelerating cancer progression (Dai E.Y. et al., 2020). Prominin 2 is a pentaspanin protein involved in lipid dynamics regulation. It promotes the formation of ferritin-containing multivesicular bodies (MVBs) and exosomes that transport iron out of the cell and thus inhibits ferroptosis (Brown et al., 2019). Exosomes themselves are also proved to have some curing functions; for instance, rat plasma-derived exosomes can enhance cell proliferation and radio-resistance-related genes and yet downregulate ferroptosis in irradiated fibroblasts (Gan et al., 2021).

### Delivery of Protein and Small RNAs

Despite the therapeutic potential of nucleic acid and protein drugs, their clinical application has been limited partly by a lack of appropriate delivery systems. Proteins and small RNAs can be loaded onto exosomes and delivered to target cells, interfering with various pathways. For example, a research team engineered human embryonic kidney (HEK) cells to produce exosomes able to target breast cancer cells overexpressing epidermal growth factor receptor (EGFR). In order to achieve elective targeting, researchers have engineered donor HEK cells to express the transmembrane domain of platelet-derived growth factor receptors fused to the GE11 peptide. Let-7a miRNA was introduced into GE11-positive exosomes using the lipofection method and HEK cells. Results show that miRNA exosomes have a curing effect on breast cancer (Ohno et al., 2013). As with ferroptosis, we reviewed a number of proteins and RNAs regulating ferroptosis-based cell death in the previous section. These molecules can be easily introduced to donor cells, and tumor targeting exosomes carrying these molecules can be used for cancer treatment. However, related research is lacking. Although the use of exosomes as a delivery system has its drawback (for example, quickly eliminating by the reticuloendothelial system, lack of efficient encapsulation methods, and potential immune responses), exosomes targeted at tumors may allow systemic administration of miRNA as cancer treatment and are thus worthy of attention.

### Advantages of Exosomes for Drug Delivery Systems

Nanotechnology has been developed for drug delivery, but intrinsic immunogenicity and residual cytotoxicity have hindered its application. During recent decades, researchers turned to delivery systems based on natural and synthetic polymers and lipids because such liposomes possess valuable qualities, such as the incorporation of hydrophilic and hydrophobic drugs, and membrane penetration (Farooqi et al., 2018). However, disadvantages, such as lower circulation stability, rapid clearance by phagocytosis, and increased toxicity, challenge the application of liposomes (Ha et al., 2016). In this respect, exosomes display better tolerance and lower toxicity due to their ubiquitous presence and similarity in structure and composition to biological membranes (Bang and Thum, 2012). Exosomes can penetrate through tissues, deliver contents directly into cellular compartments, and evade the immune system. They are also able to target specific organs and tissues (Hood et al., 2011). The application of engineered cell strains with special plasmid vectors that encode fusion proteins helps to develop targeted exosome-based delivery systems by achieving amenable membrane modifications and desirable attributes especially when targeting a specific cell type (Farooqi et al., 2018).

### Application of Exosomes Providing a Novel View for Ferroptosis-Based Cancer Treatment

Modified exosomes can be selectively used to deliver drugs to specific cells and present advantages, such as high effectiveness and reduced toxicity. Exosomes generated by genetically engineered cell stains present designed proteins on the surface, which can selectively drive exosomes and their contents to targeted cells. For example, engineered immature dendritic cells (imDCs) in mice express a well-characterized exosomal membrane protein (Lamp2b) fused to αv integrin-specific iRGD peptide (CRGDKGPDC). Doxorubicin (Dox), produced by engineering imDCs, can be loaded to exosomes through electroporation. *In vivo* and *in vitro* experiments have shown that the Dox-exosomes process possesses high efficacy in Dox delivery and targeting to breast cancer, effective cancer suppression, and low toxicity (Tian et al., 2014). One study showed that exosomes derived from brain cells that expressed brain specific surface proteins can cross the blood–brain barrier and deliver drugs to the other side (Yang et al., 2015). For ferroptosis, therapeutic drugs, such as erastin and newly recognized natural ferroptosis-inducing compounds, can be loaded onto tumor targeting exosomes. This may provide new avenues for cancer treatment. Attempts have been made and the results are positive. Nevertheless, challenging issues remain to be solved, such as poor encapsulation efficiency and the interference form exosomal endogenous nucleic acids and proteins.

## Conclusion and Future Prospect

Apoptosis, necroptosis, pyroptosis, and ferroptosis are the most widely studied types of programmed cell death. These types of programmed cell death are all involved in cancer progression and therapy. In our lab, we focus on regulating the crosstalk among different types of programmed cell death to broaden the application of anti-tumor drugs (Liu S. et al., 2017; Huang et al., 2018, 2021a,b; Xiang et al., 2019; Li T. et al., 2020). Inducing a certain type of programmed cell death specifically can have profound significance for cancer treatment. Ferroptosis is an iron-dependent form of programmed cell death triggered by unrestricted lipid peroxidation and subsequent plasma membrane rupture. It is well known that cancer development and treatment can be affected by genetic factors, epigenetic modifiers, CSCs, and the TME (Xiang et al., 2019). As mentioned above, ferroptosis could be induced to exert anti-tumor functions *via* signaling pathway modulation, non-coding RNA expression, DNA methylation, histone modification, CSCs, microenvironment remodeling, and so on. However, it is not clear how to utilize and manipulate ferroptosis in cancer treatment, specifically. On the one hand, studies need to deeply exploit the molecular and cellular mechanisms underlying ferroptosis; on the other hand, combining ferroptosis with biological materials is a promising alternative strategy. As the smallest extracellular vesicles and endogenous source of nanocarriers, exosomes show great potential for cargo delivery, including RNA, protein, drugs, and ions. Most importantly, exosomes have been shown to transport iron out of the cell to regulate ferroptosis (Brown et al., 2019). In addition, gene engineered exosomes exhibit promising characteristics in cancer treatment (Cheng et al., 2021). Therefore, adjusting the cargo of exosomes and/or engineering their spreading pathways could target cancer cells (especially CSCs) or the TME in order to induce ferroptosis, thus achieving a positive therapeutic outcome.

## Author Contributions

SW and YH wrote the manuscript. TL and WL reviewed the manuscript. All authors approved the manuscript.

## Conflict of Interest

The authors declare that the research was conducted in the absence of any commercial or financial relationships that could be construed as a potential conflict of interest.

## Publisher’s Note

All claims expressed in this article are solely those of the authors and do not necessarily represent those of their affiliated organizations, or those of the publisher, the editors and the reviewers. Any product that may be evaluated in this article, or claim that may be made by its manufacturer, is not guaranteed or endorsed by the publisher.
